# Genetic Diversity and Classification of the Cytoplasm of Chinese Elite Foxtail Millet [*Setaria italica* (L.) P. Beauv.] Parental Lines Revealed by Chloroplast Deoxyribonucleic Acid Variation

**DOI:** 10.3389/fgene.2019.01198

**Published:** 2019-11-22

**Authors:** Dan Liu, Yanjiao Cui, Jihong He, Suying Li, Qiang Li, Dan Liang, Jianhe Wang, Xiaowei Shi, Conglei Wang, Kongjun Dong, Tianpeng Liu, Lei Zhang, Ruiyu Ren, Tianyu Yang, Gang Feng, Zhengli Liu

**Affiliations:** ^1^Tianjin Crop Research Institute, Tianjin Academy of Agricultural Sciences, Tianjin, China; ^2^Department of Life Sciences, Tangshan Normal University, Tangshan, China; ^3^Crop Research Institute, Gansu Academy of Agricultural Sciences, Lanzhou, China

**Keywords:** foxtail millet, elite parental lines, genetic diversity, cytoplasmic type, chloroplast deoxyribonucleic acid

## Abstract

Due to the maternal inheritance of cytoplasm, using foxtail millet [*Setaria italica* (L.) P. Beauv.] male sterile lines with a single cytoplasmic source as the female parent will inevitably lead to a narrow source of cytoplasm in hybrids, which may make them vulnerable to infection by cytoplasm-specific pathogens, ultimately leading to destructive yield losses. To assess cytoplasmic genetic diversity in plants, molecular markers derived from chloroplast DNA (cpDNA) have been used. However, such markers have not yet been applied to foxtail millet. In this study, we designed and screened nine pairs of polymorphic foxtail millet-specific primers based on its completely sequenced cpDNA. Using these primers, we analyzed the genetic diversity and cytoplasmic types of 130 elite foxtail millet parental lines collected in China. Our results revealed that the cytoplasmic genetic diversity of these accessions was low and needs to be increased. The parental lines were divided into four cytoplasmic types according to population structure analysis and a female parent-derivative evolutionary graph, indicating that the cytoplasmic types of elite foxtail millet lines were rather limited. A principal component analysis (PCA) plot was linked with the geographic and ecological distribution of accessions for each cytoplasmic type, as well as their basal maternal parents. Collectively, our results suggest that enriching cytoplasmic sources through the use of accessions from diverse ecological regions and other countries as the female parent may improve foxtail millet breeding programs, and prevent infection by cytoplasm-specific pathogens.

## Introduction

Heterosis refers to the superior performance of hybrids compared with their parental lines and has been extensively exploited to increase grain yield in many crops for more than a century, including maize (*Zea mays* L.), sorghum (*Sorghum bicolor* L.), and rice (*Oryza sativa* L.) (Guo et al., 2006; Springer and Stupar, 2007; Baranwal et al., 2012). Foxtail millet [*Setaria italica* (L.) P. Beauv.] originated in China, and has been a traditional cereal food crop in China since ancient times. Recently, researchers have developed foxtail millet hybrid cultivars based on the heterosis theory and successfully increased yields (Huang et al., 2018; Li et al., 2018). During the development of foxtail millet hybrids, a male sterile line and a restorer line are used. China is the center of diversity for foxtail millet landraces, and to date, 27,059 accessions have been recorded in the Chinese National GeneBank (CNGB) (Diao and Jia, 2017). Despite this, male sterile lines are represented by limited genetic diversity. Pedigree analysis indicates that almost all Chinese spring foxtail millet male sterile lines are derived from Chang10A, with cytoplasm from Qinyuanmujizui (Wang et al., 1998; Liu et al., 2011). Similarly, most Chinese summer foxtail millet male sterile lines are derived from Huangmi1A, which contains cytoplasm from Dahuanggu (Liu et al., 1996; Liu et al., 2006).

The wide use of a single source of cytoplasm presents considerable risks to agricultural production. For example, in the 1970s in the United States, the widespread use of maize Texas male-sterile cytoplasm (*cms*-T) rendered hybrid cultivars susceptible to the fungal pathogen *Helminthosporium maydis*, which caused an outbreak of southern leaf blight and resulted in a serious yield loss (Hooker et al., 1970; Scheifele et al., 1970; Smith et al., 1970). In 1984, the widespread use of Xianyou 2 in China, a wild abortion type rice male sterile cytoplasm, resulted in a major epidemic of *Pyricularia oryzae*, representing one of the most devastating rice fungal diseases (Liu et al., 1992; Yuan, 1997). Currently, the primary male sterile cytoplasms of foxtail millet cultivars in China are Qinyuanmujizui and Dahuanggu. Due to the maternal inheritance of cytoplasm, hybrid cultivars produced by using these male sterile lines still carry the same source of cytoplasm. Long-term and large-scale use of a single source of cytoplasm in foxtail millet male sterile lines may eventually cause infections by certain pathogens and ultimately lead to destructive losses in foxtail millet hybrid production. Thus, it is extremely important to enrich the cytoplasm resources in male sterile lines, in particular through a better understanding of the genetic diversity and classification of Chinese foxtail millet cytoplasms.

In recent years, molecular markers have been used to assess the genetic diversity of foxtail millet. To investigate the genetic diversity and population structure, Liu et al. (2011) first screened a collection of 128 foxtail millet germplasm and elite breeding lines from three major ecological areas of China for foxtail millet growth using 79 SSR markers. Subsequently, researchers conducted population diversity and structure analyses of Chinese foxtail millet landraces and cultivars using microsatellite markers (Wang et al., 2012a; He et al., 2015; Jia et al., 2015). However, these studies mainly focused to nuclear genomes. As such, there is a scarcity of reports on the genetic diversity of foxtail millet cytoplasm.

Both types of cytoplasmic genomes, mitochondrial DNA (mtDNA) and chloroplast DNA (cpDNA), represent important genetic material in addition to the nuclear genome of plants. Their distinct features, including small genome sizes, high conservation, and the absence of sexual recombination make mtDNA and cpDNA useful tools for determining phylogenetic relationships and studying plant populations (Avise, 2000; Provan et al., 2001; Norouzi et al., 2012; Cheng et al., 2015; Skuza et al., 2019). Recently, molecular markers derived from cpDNA and mtDNA have been designed and used for research on cytoplasmic variation in soybean (*Glycine max*) (Shimamoto et al., 2000), rice (Rajendrakumar et al., 2007), *Lolium* species (McGrath et al., 2007), and *Brassica* species (Wang et al., 2012b; Zamani-Nour et al., 2013; Shu et al., 2016). However, genetic diversity analysis and classification of cytoplasm types in foxtail millet accessions with molecular markers based on cpDNA or mtDNA have rarely been reported. In a previous study, we analyzed the genetic diversity of the cytoplasm of 111 elite foxtail millet breeding lines using 23 mtDNA consensus primer pairs, and constructed a phylogenetic tree to classify these accessions (Liu et al., 2014). However, the genetic diversity analysis and classification of elite parental lines have not yet been investigated, but is crucial for creating male sterile lines that carry new cytoplasm types.

In this study, the cytoplasmic genetic diversity of 130 elite foxtail millet parental lines was assessed based on cpDNA polymorphism. We used nine foxtail millet-specific primer pairs, which were developed based on the completely sequenced chloroplast genome. To classify the cytoplasmic types of these elite parental lines, we used population structure analysis and female parent-derivative evolutionary graphing. We aimed to understand the relationship between the geographic and ecological distribution of the tested accessions and their basal maternal sources by using principal component analysis (PCA). Our results provide insight for enriching the cytoplasmic types of foxtail millet hybrids in China and around the world, and also provide a reference to broaden the cytoplasmic types of conventional cultivars and hybrids of other crops.

## Materials and Methods

### Plant Materials

A total of 130 representative Chinese elite foxtail millet parental lines were collected. These materials were sourced from diverse geographic origins that spanned three ecotypes (north summer millet, northwest spring millet, and northeast spring millet). These accessions were primarily from eight foxtail millet growing provinces and cities (1 accession in Beijing, 107 accessions in Hebei, 3 accessions in Henan, 12 accessions in Shanxi, 1 accession in Shandong, 2 accessions in Jilin, 3 accessions in Liaoning, and 1 accession in Shaanxi), where the majority of Chinese foxtail millet breeding programs are located. Among the 130 elite parental lines, 51 accessions were developed and released after 2014. Information on the collected samples is summarized in **Supplemental Table S1**.

### Deoxyribonucleic Acid Extraction, Primer Definition, and Selection

The cpDNA of four foxtail millet accessions (Gu56A, Gu572A, Datong28, and Datong29lv) was extracted and sequenced as described in a previous study (unpublished data, the chloroplast genome sequences have been deposited to GenBank). Genomic DNA used for DNA polymorphism detection was extracted using the DNAquick Plant System DP321 (Tiangen Biotech, Beijing, China) following the manufacturer’s instructions.

Single nucleotide polymorphisms (SNPs) and insertion-deletion polymorphisms (InDels) in four cpDNAs (Gu56A, Gu572A, Datong28, and Datong29lv) were detected by comparing the aligned sequences in DNAMAN software to identify those conducive for genotyping. Twenty-seven primer pairs were designed to amplify these regions and were selected to screen the genomes of 130 foxtail millet accessions. A total of nine primers with high levels of polymorphisms were used for further analysis. Primer sequences, annealing temperatures for PCR amplification, and amplification product sizes are summarized in **Table 1**.

**Table 1:** Information on the nine chloroplast markers used in this study.

Name	Forward sequence (5’-3’)	Reverse sequence (5’-3’)	Location	Type	Tm (°C)	Product size (bp)
Ch-2F/1R	GCGGAAGCCCGTTTATAC	AAAAAGATGGTACTAGCGAAA	*rpo*C1-*rpo*C2	IGS + Gene	53	610
Ch-3F/3R	CTCCTGTCTCCCGCTATT	AGTCAGTGATTCGAAAACAAA	*rpo*C1-*rpo*C2	IGS + Gene	53	620
Ch-6F/6R	CAGAAAAGAGAGGATAGAGGATAG	CACCAAGACCTATAATACGAGC	*atp*F-*atp*A	Intron + Exon	53	822
Ch-8F/8R	GTTGGATCATAGTCTCTTACACG	ACGATTTCGATAGTCATACCG	*psa*A	Exon	49	1,090
Ch-9F/8R	GTTGGATCATAGTCTCTTACCGA	ACGATTTCGATAGTCATACCG	*psa*A	Exon	57	1,090
Ch-10F/8R	AATTCAAGGTGAAAAAGCCA	ACGATTTCGATAGTCATACCG	*psa*A	Exon	55	500
Ch-12F/15R	ACCCAGAAATATACATACCCAG	CGATCCTACTCACATTCCA	*psa*A	Exon	57	1,030
Ch-20F/17R	TCCATGAAGTAAGACATTGATAAT	TGAGTAGAGCTGAGGGTACGA	*atp*E-*ndh*C	IGS + Gene	51	621
Ch-27F-1/23R	CTATAGGTTGTGTCGTATCACAT	AAGCCAGTTTCAACAATACC	*rps*11-*rpo*A	IGS + Gene	53	784

A set of 42 universal cpDNA primers (https://www.ncbi.nlm.nih.gov/probe/?term=chloroplast+ssr) and 34 mtDNA consensus primers (Duminil et al., 2002) were selected to amplify the target sequences of genomes from the 130 selected foxtail millet accessions. Ten cpDNA primers and 17 mtDNA primers were used in this study according to their high levels of polymorphisms across these accessions. Primer sequences, annealing temperatures for PCR amplification, and amplification product sizes are summarized in **Supplemental Tables S2** and **S3**.

### Polymerase Chain Reaction Amplification and Gel Electrophoresis

PCR amplification was conducted in 10 µl reaction mixtures, which contained 20 ng template DNA, 10 pmol of each forward and reverse primers, and the 2× Taq PCR Master Mix (Tiangen Biotech, Beijing, China). The reaction was carried out with a thermocycler (Biometra, Göttingen, Germany) using the following conditions: an initial denaturation step of 95°C for 5 min, followed by 35 cycles of 95°C for 30 s, an appropriate annealing temperature of different primers (**Table 1** and **Supplemental Tables S2** and **S3**) for 30 s, 72°C for 2 min, and a final elongation step of 72°C for 10 min.

Amplified products were separated on a 1.5% agarose gel in 1×TAE buffer along with the DNA ladder DL2000 (Sangon, Shanghai, China) as a size marker. Next, the PCR products were visualized and photographed under a Gel Dox XR+ (Bio-Rad, Madison, CA, USA) photography system using ultraviolet light. Data were scored manually for band presence or absence and entered into a matrix for further analysis.

### Data Analysis

Summary statistics, including the number of alleles per locus, major allele frequency, gene diversity, and polymorphism information content (PIC) values were determined using PowerMarker v3.25 (Liu and Muse, 2005). To detect population genetic structure and assign individuals to subpopulations, the program STRUCTURE v2.3.4 (Pritchard et al., 2000; Falush et al., 2003) was used, which employs a Bayesian clustering approach. Ten independent runs for cluster (*K*) values, which ranged from 2 to 10, were performed after a burn-in period of 5 × 10^5^ Markov Chain Monte Carlo steps, followed by 1 × 10^5^ replicates using the admixture model. The output was exported into Structure Harvester (Earl and vonHoldt, 2012) to determine the most likely number of *K* clusters (*K* = 4 was optimum for this analysis) using Evanno’s Δ*K* method (Evanno et al., 2005). Results from 10 independent STRUCTURE runs for the most likely *K* were assessed with the software CLUMPP (Jakobsson and Rosenberg, 2007) and plotted using the DISTRUCT program (Rosenberg, 2010).

Arlequin 3.11 (Excoffier et al., 2005) was used to conduct the analysis of molecular variance (AMOVA) and to calculate the pairwise *F*_ST_ values. A PCA was performed with SPSS 17.0 for Windows (SPSS Inc., Chicago, USA) using standard statistical methods.

## Results

### Designing of Chloroplast Deoxyribonucleic Acid Primer Pairs

Prior to this study, cpDNAs of four different foxtail millet accessions (Gu56A, Gu572A, Datong28, and Datong29lv) were extracted and sequenced. In this study, 27 primer pairs were designed and used for PCR amplification using the total DNA from 130 accessions. Primer pairs were designed based on the SNPs and insertion-deletion polymorphisms (InDels) in the foxtail millet cpDNAs. Nine pairs showed polymorphisms and were selected according to preliminary screening, with the percentage of polymorphism accounting for 33.33%. These primer pairs were all located in the large single copy (LSC) region of the foxtail millet chloroplast genome, and amplified coding (exon 44%) and mixed regions [exon + intron 12%, Intergenic Spacers (IGS) + gene 44%].

After genotyping the 130 foxtail millet accessions using the nine chloroplast DNA markers, a total of 20 alleles were detected. There was an average of two alleles per locus and the average major allele frequency was 0.70. The PIC values ranged from 0.03 to 0.61, with an average of 0.30. The mean gene diversity was 0.37 (**Table 2**). These results indicated that the cytoplasmic genetic diversity of foxtail millet accessions assessed here was low.

**Table 2:** Data summary for 130 foxtail millet accessions based on nine chloroplast DNA markers.

Marker	Major allele frequency	Sample size	No. of alleles	Gene diversity	PIC
2F/1R	0.52	130	2	0.50	0.37
3F/3R	0.56	130	2	0.49	0.37
6F/6R	0.65	130	2	0.45	0.35
8F/8R	0.81	130	2	0.31	0.26
9F/8R	0.98	130	2	0.03	0.03
10F/8R	0.89	130	2	0.19	0.17
12F/15R	0.58	130	2	0.49	0.37
20F/17R	0.91	130	2	0.17	0.15
27F-1/23R	0.41	130	4	0.67	0.61

### Population Structure Analysis

**Figure 1:**
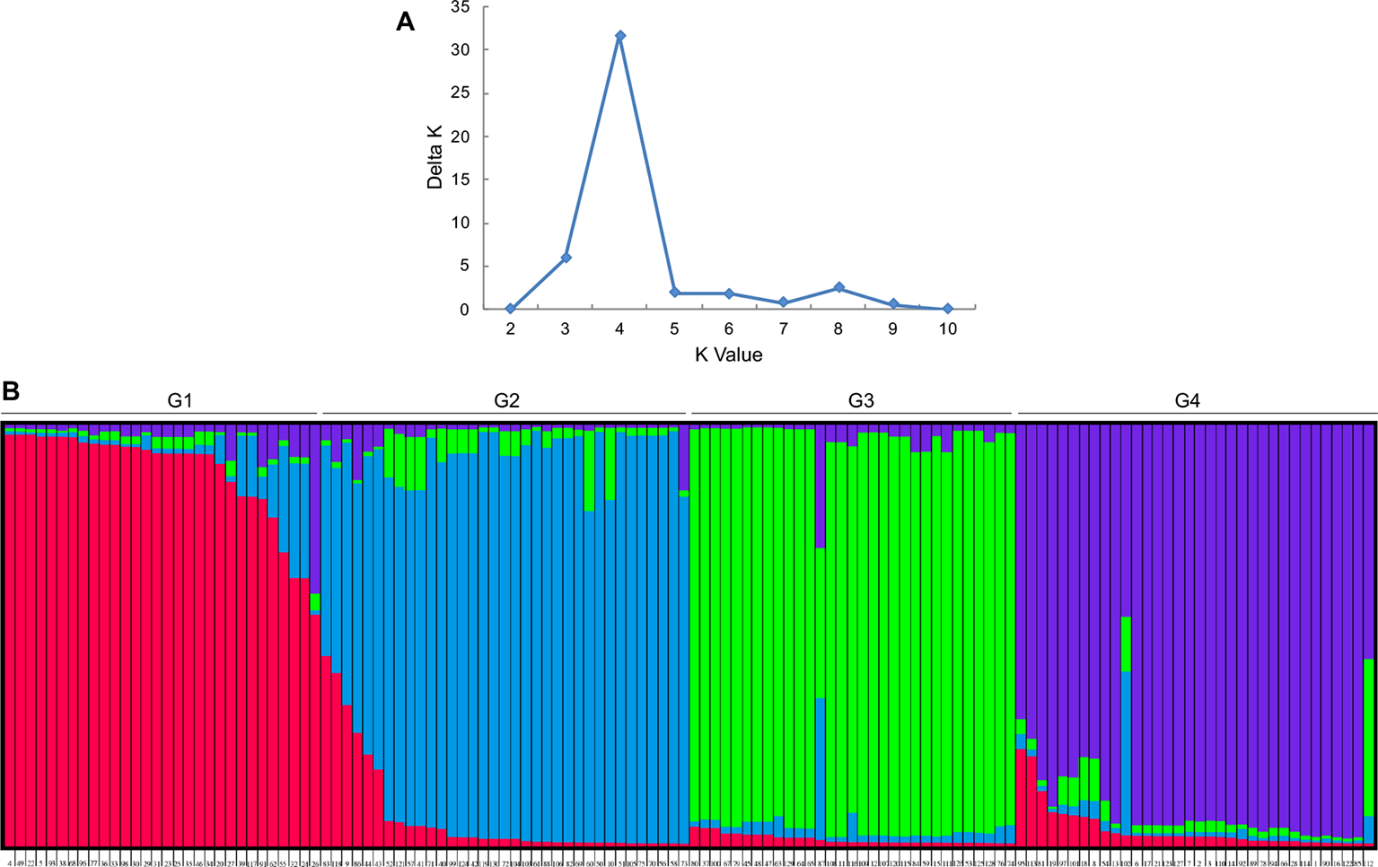
Population structure analysis for 130 accessions of foxtail millet. **(A)** Delta *K* values for different numbers of populations assumed (*K*) in the structure analysis. **(B)** Classification of 130 accessions into four subpopulations according to preset *K* value using STRUCTURE program. The distribution of the accessions to different subpopulations is indicated by color (G1: red, G2: blue; G3: green; G4: purple).

An analysis of the population structure of the 130 foxtail millet accessions showed that the most appropriate grouping was four subpopulations with a Δ*K* peak (*K* = 4 using the program STRUCTURE; **Figure 1A**). Thus, the group of foxtail millet accessions were divided into four subpopulations termed G1, G2, G3, and G4 (**Figure 1B**). Among the four subpopulations, the level of genetic diversity within G2 was the highest (0.28), followed by G1 (0.26), G4 (0.25), and G3 (0.23). Additionally, G2 also had the most alleles scored (**Table 3**).

**Table 3:** Summary statistics for subpopulations detected by structure analysis based on nine chloroplast DNA markers.

Statistics	G1	G2	G3	G4
Sample size	30	35	31	34
No. of alleles	1.78	2.00	1.67	1.78
Major allele frequency	0.80	0.80	0.82	0.81
Gene diversity	0.26	0.28	0.23	0.25
PIC	0.21	0.23	0.17	0.20

An analysis of molecular variance (AMOVA) was performed to estimate the genetic variance among and within populations. The test showed that the genetic variation among individuals within subpopulations was 65.20%, higher than that between subpopulations, which was 34.80% (**Table 4**). The pairwise *F*_ST_ analysis indicated that the genetic distance between G2 and G3 was the smallest (0.25646), and the genetic distance between G2 and G4 was the largest (0.42528) (**Table 5**).

**Table 4:** The analysis of molecular variance (AMOVA) among and within four populations of 130 foxtail millet accessions identified by structure analysis.

Source of variation	Sum of squares	Variance components	Percentage of variation
Among populations	130.534	0.63373	34.80
Within populations	299.158	1.18714	65.20
Total	429.692	1.82086	

**Table 5:** Pairwise *F*_ST_ among four populations of 130 foxtail millet accessions identified by structure analysis.

Group	G1	G2	G3	G4
G1	0.0000			
G2	0.37453	0.0000		
G3	0.40537	0.25646	0.0000	
G4	0.30036	0.42528	0.34933	0.0000

### Construction of a Female Parent-Derivative Evolutionary Graph and Classification of Cytoplasm Types

**Figure 2:**
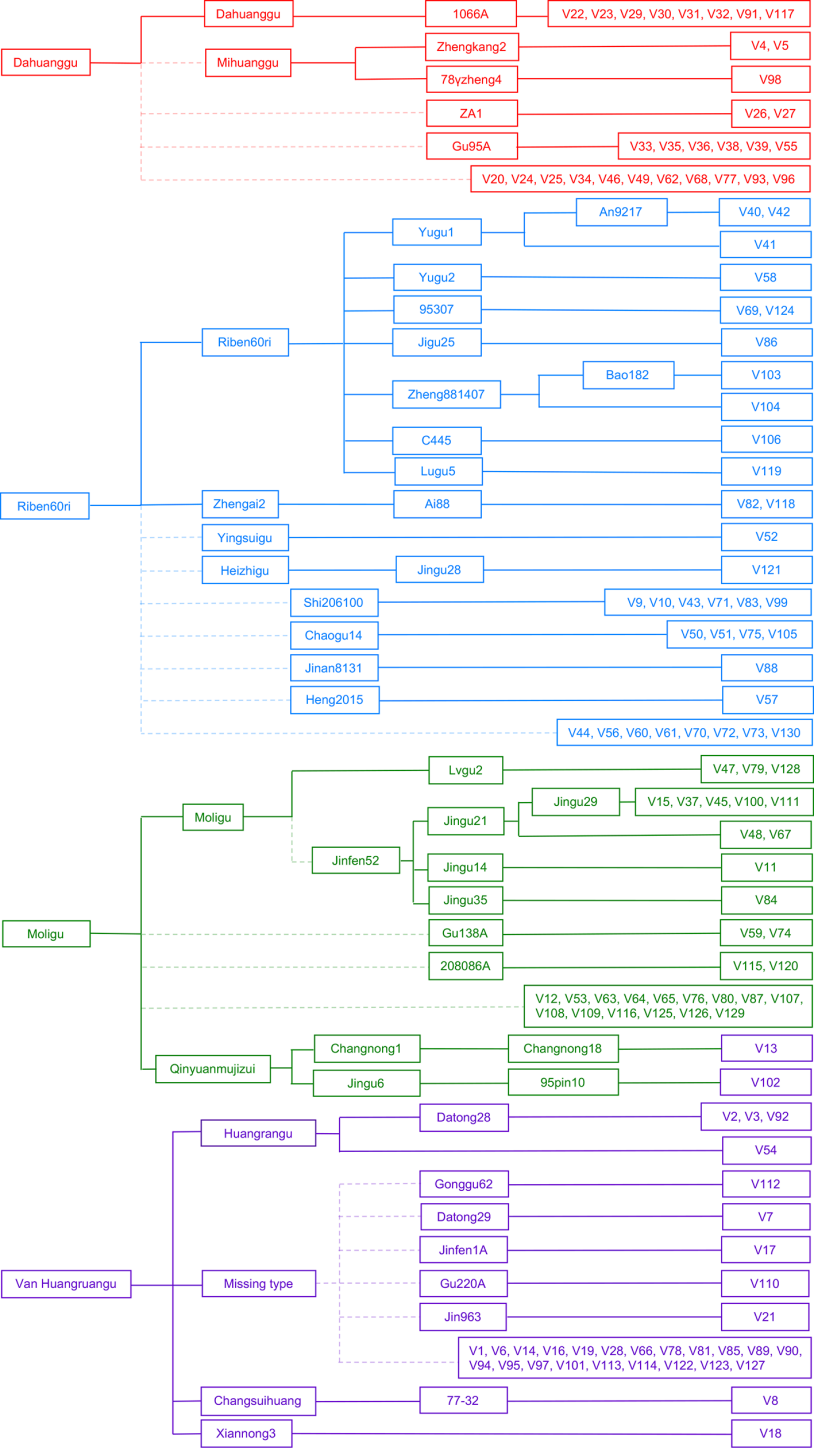
Classification of the cytoplasm types of the 130 foxtail millet accessions based on the female parent-derivative evolutionary graph and population structure analysis. The colors represent population membership identified using STRUCTURE in **Figure 1**.

We constructed a female parent-derivative evolutionary graph of the 130 accessions according to their maternal genealogy, and clustered them into four cytoplasmic types by combination with the results of population structure analysis (**Figure 2**). Type 1 (G1) contained 30 accessions; 8 were Dahuanggu derivatives, 3 were Mihuanggu derivatives, and 19 were from unknown maternal sources. As Dahuanggu and Mihuanggu both originated from Henan Province in China and are classified into the G1 group, we speculated that Dahuanggu and Mihuanggu were the same accession. Thus, the G1 type likely has the same cytoplasm as Dahuanggu, and can be designated as a Dahuanggu type. A total of 35 accessions were grouped in type 2 (G2), including 11 Riben60ri derivatives, two Zhengai2 derivatives, one Yingsuigu derivative, one Heizhigu derivative, and 20 accessions with unknown pedigrees. Since Zhengai2 is a derivative of Riben60ri (Wei et al., 1998), and the majority of the accessions in the G2 group (86.7%) were from Riben60ri, their cytoplasm type is considered as Riben60ri type. Type 3 (G3) consisted of 31 accessions; 12 were Moligu derivatives, and the remaining 19 accessions were from unknown maternal sources. The G3 group fully matches the Moligu cytoplasm type and was designated as a Moligu type. The remaining 34 accessions were categorized into type 4 (G4). Type 4 was a mixed subpopulation, including 4 Huangruangu derivatives, 2 Qinyuanmujizui derivatives, 1 Xiannong3 derivative, 1 Changsuihuang derivative, and 26 accessions having unknown pedigrees. Since half the accessions in the G4 group (50%) were from Huangruangu, their cytoplasm type is defined as Van Huangruangu type.

### Geographic and Ecological Distribution of Different Cytoplasmic Types

**Figure 3:**
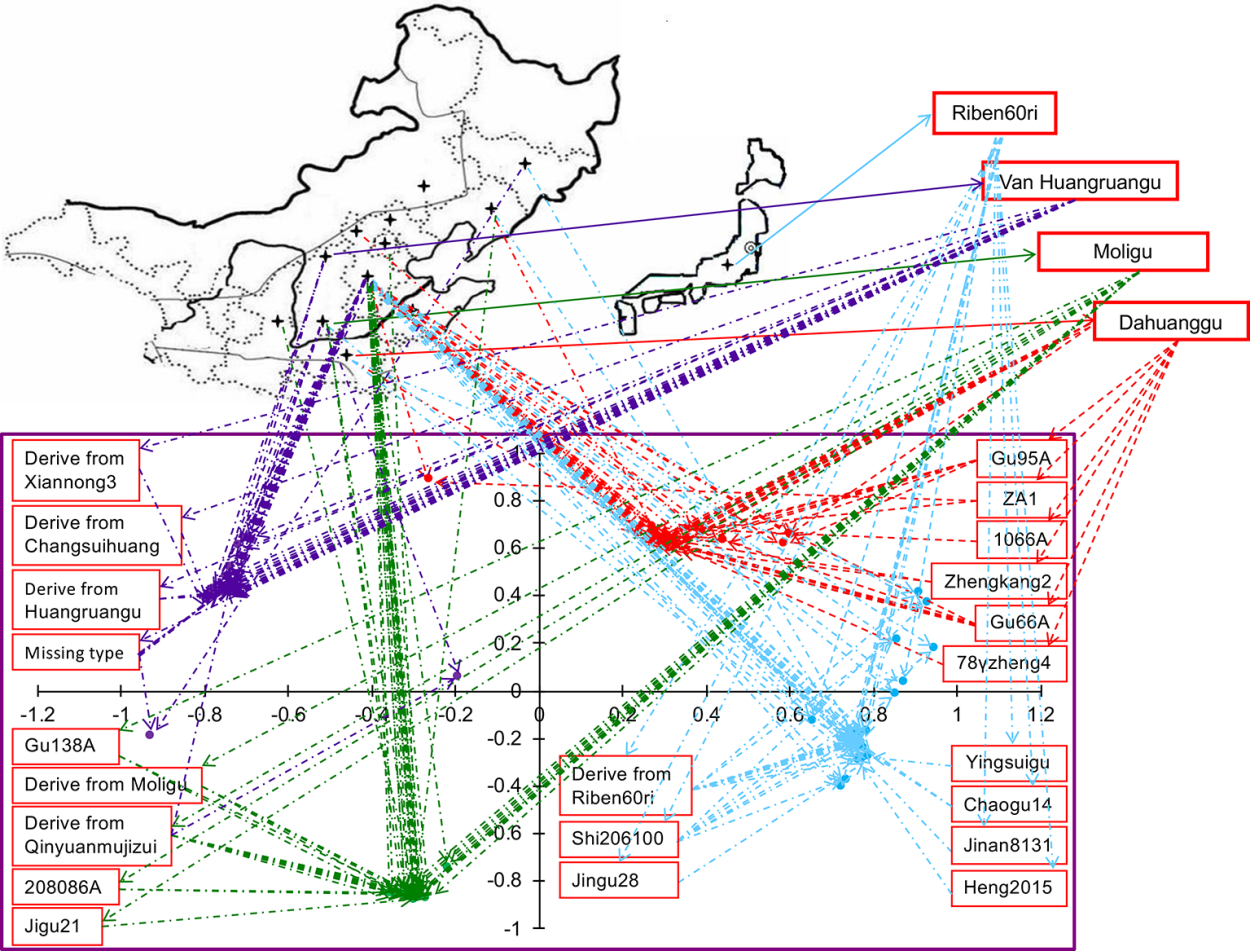
Geographical and genetic distributions of 130 accessions of foxtail millet. The lower plot is from the principal component analysis (PCA). The lines link the position of accessions in the PCA graph and their geographic origin on the map. The colors indicate population membership identified using STRUCTURE in **Figure 1**.

When we linked sample position on the two-dimensional PCA plot to the geographic distribution on the world map, we found that the accessions of same cytoplasmic type were closely related genetically, however, their geographic and ecological distributions were not the same (**Figure 3**). Among the 30 accessions of the Dahuanggu type (G1), 27 (90.01%) accessions were from central and south Hebei Province, and one each (3.33%) was from Henan Province, Liaoning Province, and the northwest Hebei Province. Except for one accession from the northwest Hebei Province (3.33%) belonging to the northwest spring millet ecotype, and one accession from Liaoning Province (3.33%) belonging to northeast spring millet ecotype, all other accessions (93.34%) were cultivated in the north China summer millet region. Among the Riben60ri type (G2), 29 (82.86%) accessions were from central and south Hebei Province, two (5.70%) were from Henan Province, and one each (2.86%) was from Jilin, Liaoning, Shanxi, and Shandong Provinces. These accessions mainly belong to three different ecological ecotypes: the north summer millet ecotype (91.43%), the northwest spring millet ecotype (2.86%), and the northeast China spring millet ecotype (5.71%).

Among the 31 accessions in the Moligu type (G3), 26 (83.87%) were from Beijing and central and south Hebei Province, and belonged to the north China summer millet ecotype. Four (12.90%) accessions were from Shanxi and Shaanxi Province and were grown in the northwest China spring millet region. One (3.23%) accession from Liaoning Province was distributed in the northeast China spring millet region. Among the Van Huangruangu type (G4), 25 (73.53%) accessions from central and south Hebei Province were grown in the north China summer millet region, eight accessions (23.53%) from Shanxi Province were distributed in the northwest China spring millet region, and one (2.94%) accession belonging to the northeast China spring millet ecotype was from Jilin Province. It is clear that the majority of the elite foxtail millet parental lines of each cytoplasmic type belong to the north China summer millet ecotype, although they have different geographic distributions.

We also focused on the distribution of basal maternal sources of the 130 tested accessions. The basal maternal parent of accessions in the Dahuanggu type (G1), from Henan Province, belonged to the north China summer millet region. Riben60ri, which was the basal maternal source of accessions in the Riben60ri type (G2), was originally introduced from Japan and is widely used in the north China summer millet region. The ecological distribution of Dahuanggu and Riben60ri coincided with the distribution of most of the accessions in the Dahuanggu (G1) and Riben60ri (G2) types. By contrast, accessions in the Moligu (G3) and Van Huangruangu (G4) types could be traced to the maternal parents Moligu and Huangruangu, respectively, both of which were from Shanxi Province and belonged to the northwest China spring millet ecotype. This was inconsistent with the ecological distribution of most of their derivatives.

## Discussion

Plant organelle genomes (e.g., cpDNA and mtDNA) have numerous important applications in phylogenetic and population studies. However, the sequence information of organelle genomes for many plant species remains unclear, and cpDNA or mtDNA of high purity and yield are usually difficult to extract due to nuclear DNA interference. In a previous study, we developed a new protocol and successfully isolated high-quality cpDNAs for whole-genome sequencing from four different foxtail millet accessions (Gu56A, Gu572A, Datong28, and Datong29lv) (unpublished data). According to the sequence alignment, 45 SNPs and 9 InDels were identified, which provided the most direct sequence information for the development of molecular markers based on cpDNA. This information also ensured that the PCR products that were amplified with these markers were from the foxtail millet chloroplast genomes.

To our knowledge, this is the first study that utilized foxtail millet-specific chloroplast DNA primer pairs. Moreover, through screening, nine primers with polymorphisms were selected from 27 primers and were used for cytoplasmic variation analysis. The match rate between the population structure analysis and the female parent-derivative evolutionary graph was 86.67% (**Figures 1** and **2**). These results revealed that the nine cpDNA markers that we developed were sufficient for the classification of the cytoplasmic types. Our markers may also help create new cytoplasm sources for male sterile lines and enrich the cytoplasmic types of foxtail millet hybrids.

We also analyzed the foxtail millet cytoplasmic genetic diversity with 10 universal cpDNA and 17 mtDNA primer pairs with polymorphisms. All 130 foxtail millet accessions were classified into 3 cytoplasmic types, and the coincidence rates between the population structure and the female parent-derivative evolutionary graph were 35.56 and 42.22%, respectively (**Supplemental Figures S1** and **S2** and **Supplemental Tables S4** and **S5**). These rates were much lower than the rate when specific chloroplast DNA primers were used. The cpDNA universal primers were developed based on complete chloroplast genomes of rice, *Arabidopsis thaliana*, and olive (*Olea europaea* L.) (https://www.ncbi.nlm.nih.gov/probe/?term=chloroplast+ssr). The set of mtDNA consensus primers were designed based on the fully sequenced mitochondrial genomes of *Arabidopsis thaliana* and *Beta vulgaris* (Duminil et al., 2002). Thus, it is possible that some DNA fragments that were amplified by these universal primers may not be from the cytoplasmic genome of foxtail millet. Furthermore, the genetic diversity was less informative than that revealed using specific cpDNA markers based on chloroplast genome sequences (Chaïr et al., 2005; Scarcelli et al., 2011). These results further suggest that the specific cpDNA primers we reported were more suitable for genetic variation, population analysis, and the classification of cytoplasmic types of foxtail millet.

Nuclear DNA microsatellite markers have been previously used in genetic diversity and population structure analysis for many crops, including 79 and 77 markers for foxtail millet (Liu et al., 2011; Wang et al., 2012a), 10 markers for wild rice (*Oryza rufipogon* Griff.) (Zhou et al., 2003), 96 markers for maize (Vigouroux et al., 2008), and 95 markers for sorghum (Wang et al., 2009). Along with the sequencing of the chloroplast genome, molecular markers based on cpDNA have been applied to population genetics and evolutionary studies in plants (Provan et al., 2001). In this study, nine chloroplast DNA markers specific to foxtail millet were used for population structure analysis. This number is similar to other studies in which chloroplast microsatellite markers were developed, such as Canary Island pine (*Pinus canariensis* C. Sm.) (six markers) (Gomez et al., 2003), wild soybean (*Glycine soja* Sieb. Et Zucc) (five markers) (He et al., 2012), and oilseed rape (*Brassica napus* L.) (14 markers) (Zamani-Nour et al., 2013). The size of circular DNA in the chloroplast ranges from 120 to 160 kilobases (kb) in most plants; this is much less than that the nuclear genome (Sugiura, 1992; Sugiura, 1995; Jansen et al., 2005). Thus, the number of cpDNA markers used in our research is sufficient for examining cytoplasmic genetic variation and population structure of foxtail millet accessions.

In this study, population structure analysis revealed the genetic relationships among different foxtail millet parental lines. Based on our results, we divided the 130 tested accessions into four groups. Most accessions in each group had highly similar genetic background (**Figure 1**). This resulted from the maternal inheritance mode of cpDNA (Shaver et al., 2006; Ravi et al., 2008) and further demonstrated that our population structure determination was reliable.

We detected a high level of polymorphism in the 9 chloroplast DNA markers across 130 foxtail millet accessions (**Table 2**). The mean gene diversity (0.37) and mean PIC value (0.30) were greater than in a previous report (0.29 and 0.23, respectively), which were identified using 23 consensus mtDNA primers across 111 accessions (Liu et al., 2014). This might be due to the fact that we used a more diverse selection of samples containing some accessions developed and released in the last few years (**Supplemental Table S1**), as indicated in our PCA analysis results (**Figure 3**). Before 2010, breeders primarily used locally adapted material as the female parent in order to develop new foxtail millet cultivars. This led to a narrow source of cytoplasm, which is illustrated in a previous study by Liu et al. (2014).

We found that the cytoplasmic types of Chinese foxtail millet are gradually diversifying. For example, among the 31 accessions in the Moligu type (G3), 83.87% accessions were grown in the north China summer millet region, while their basal maternal source, Moligu, was from Shanxi Province, which belongs to the northwest China spring millet region (**Figure 3**). This indicated that a greater number of foxtail millet male sterile lines were being used as the female parent cross-ecological regions in recent years. However, on the whole, the cytoplasm of Chinese foxtail millet is still of limited genetic variation, and the utilization of the foxtail millet germplasm is still primarily limited to the ecological distribution they belonged to originally. For example, the majority of accessions in Dahuanggu (G1) and Riben60ri (G2) types (93.34 and 91.43%, respectively) are cultivated in the north China summer millet region, and their basal maternal sources, Dahuanggu and Riben60ri, are also from (or widely applied) in the same ecological region (**Figure 3**). Thus, to avoid risks and losses from using of single source of cytoplasm in foxtail millet male sterile lines, finding and developing diverse cytoplasm types by using female parents introduced from different ecological regions and foreign countries is still key for improving hybrid cultivars.

It is worth noting that the Van Huangruangu type (G4) was a mixed subpopulation, which contained 25% Qinyuanmujizui derivatives, 12.5% Xiannong3 derivatives, 12.5% Changsuihuang derivatives, and 50% Huangruangu derivatives (**Figure 2**). This suggested that the accessions in the Van Huangruangu type (G4) may be derived from more than one single cytoplasmic type. Thus, the Van Huangruangu type (G4) may be a source for elite parental lines that contains different cytoplasmic types, and may therefore be used in foxtail millet breeding.

## Data Availability Statement

The datasets generated for this study can be found in the GenBank under the following accession numbers: 56A (MK348603), 572A (MK348609), Datong28 (MK348605), and Datong29lv (MK348604).

## Author Contributions

ZL and SL contributed to the study conception and design. TY, XS, CW, TL, and QL collected the foxtail millet materials. DLiu designed the cpDNA primers. SL, DLiu and QL genotyped the accessions, and determined the summary statistics. DLiu, DLia, and JW performed the population structure analysis, conducted the analysis of molecular variance, and calculated the pairwise *F*_ST_ values. JH and YC constructed the female parent-derivative evolutionary graph. The principal component analysis was performed by GF and DLiu. KD, RR, and LZ coordinated the experiments and data analysis. The first draft of the manuscript was written by YC. ZL and YC revised the article. All authors read and approved the final manuscript.

## Funding

This work was supported by National Natural Science Foundation of China [grant number 31560094].

## Conflict of Interest

The authors declare that the research was conducted in the absence of any commercial or financial relationships that could be construed as a potential conflict of interest.
