# Supplementary figures and images for: Genetic Diversity and Classification of the Cytoplasm of Chinese Elite Foxtail Millet [Setaria italica (L.) P. Beauv.] Parental Lines Revealed by Chloroplast Deoxyribonucleic Acid Variation
Source: Front Genet. 2019 Nov 22;10:1198. doi: 10.3389/fgene.2019.01198 (PMC6882946; doi:10.3389/fgene.2019.01198)

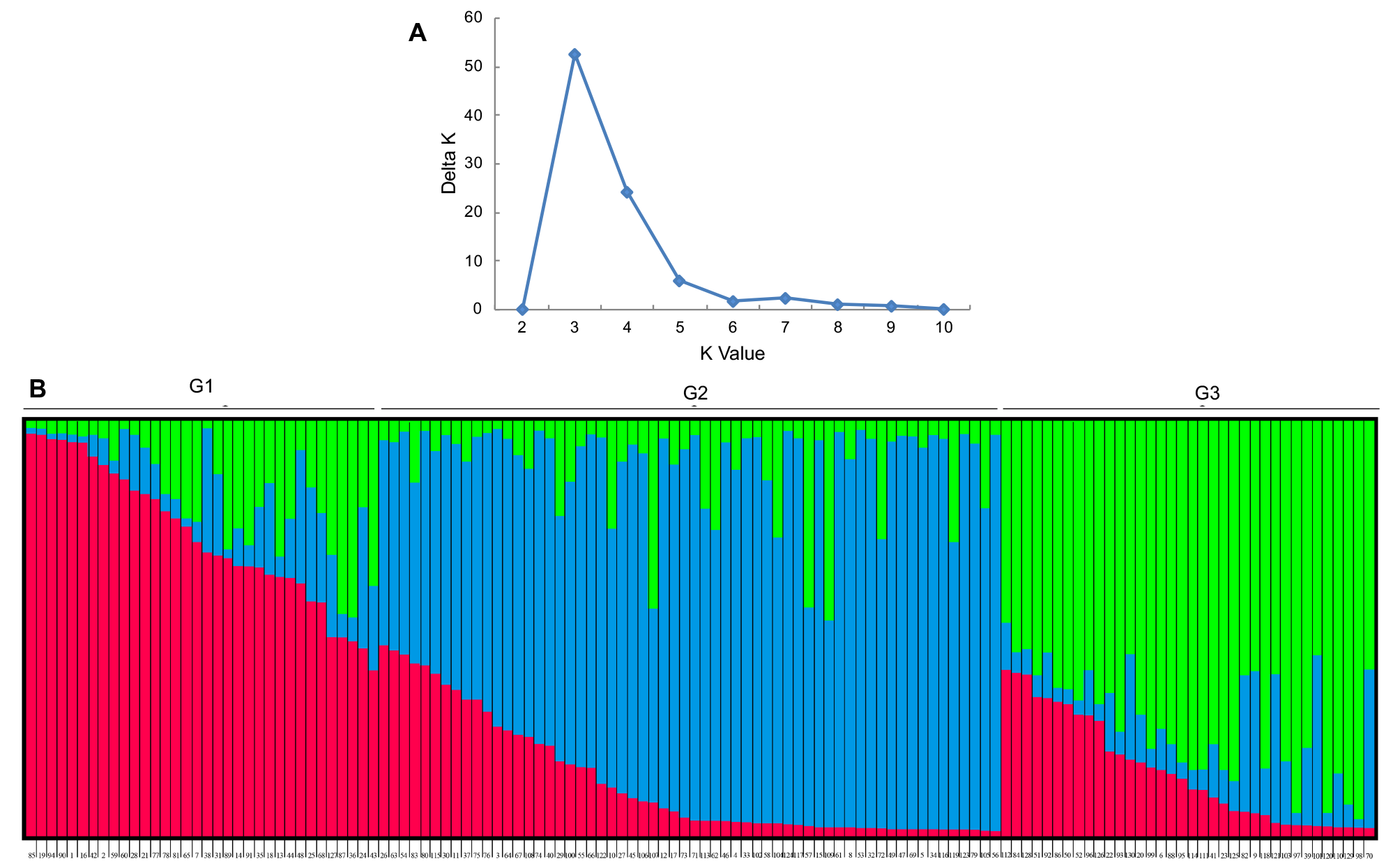

Supplement: Supplementary file 1 [file DataSheet_1.zip › Fig S1.tif]

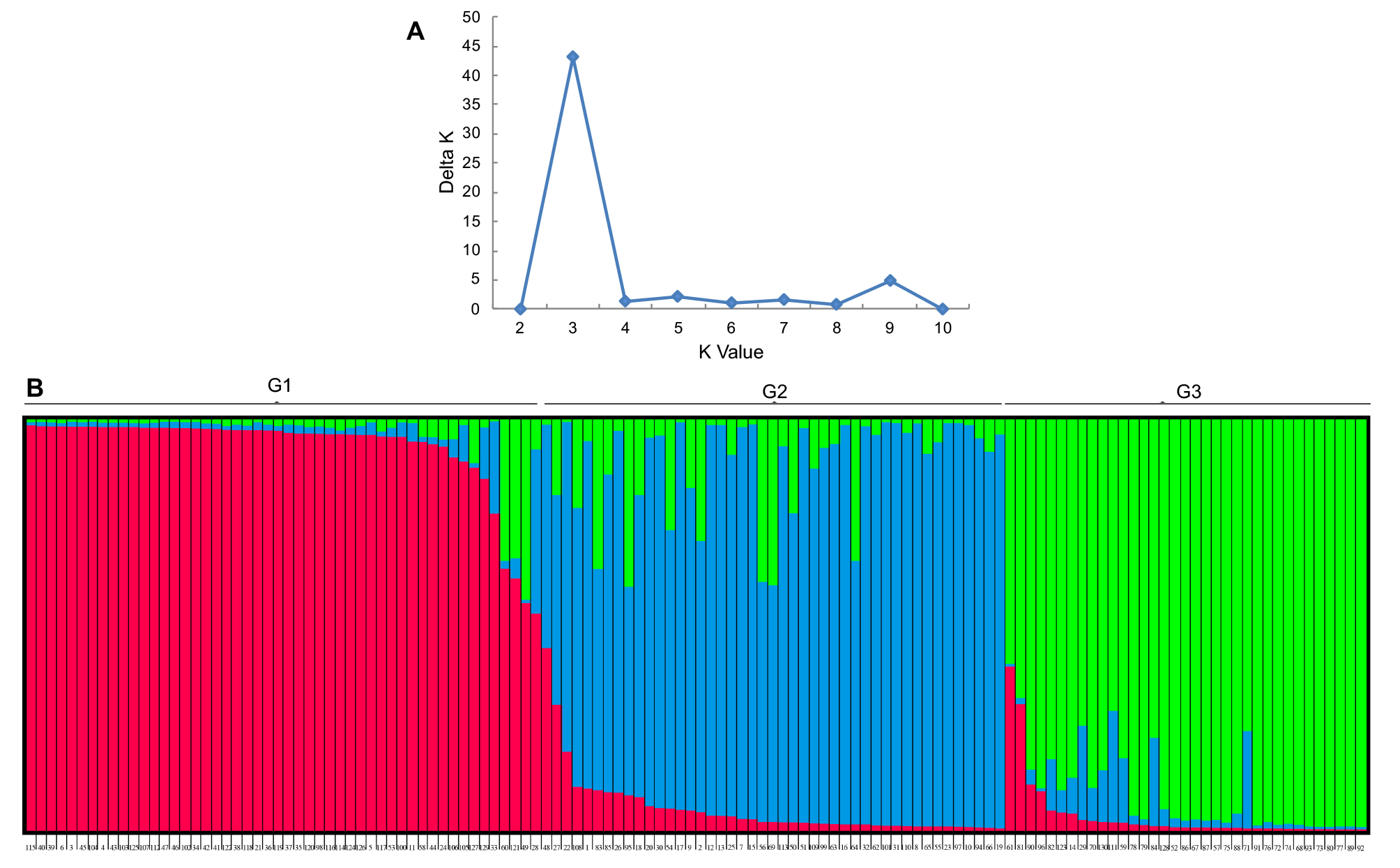

Supplement: Supplementary file 1 [file DataSheet_1.zip › Fig S2.tif]
