# Supplementary material for: Genetic Diversity and Classification of the Cytoplasm of Chinese Elite Foxtail Millet [Setaria italica (L.) P. Beauv.] Parental Lines Revealed by Chloroplast Deoxyribonucleic Acid Variation
Source: Front Genet. 2019 Nov 22;10:1198. doi: 10.3389/fgene.2019.01198 (PMC6882946; doi:10.3389/fgene.2019.01198)
Supplement: Supplementary file 1 [file DataSheet_1.zip › Supplementary_Material.doc]

Supplementary Material

**Table S1** The 130 foxtail millet accessions included in experiments and their subpopulation membership coefficients. Asterisks indicate the accessions were created and released after 2014.

| No. | Variety | Maternal source | Basal germplasm | Geographic distribution | Ecological distribution | G1 | G2 | G3 | G4 |
| --- | --- | --- | --- | --- | --- | --- | --- | --- | --- |
| 1 | Gu51A | . | . | Heibei | North summer | 0.0091 | 0.0050 | 0.0080 | 0.9779 |
| 2 | DZ234A* | Datong28 | Huangruangu | Heibei | North summer | 0.0229 | 0.0105 | 0.0258 | 0.9408 |
| 3 | KM182A* | Datong28 | Huangruangu | Heibei | North summer | 0.0228 | 0.0106 | 0.0268 | 0.9398 |
| 4 | DZ386A* | Zhengkang2 | Mihuanggu | Heibei | North summer | 0.9770 | 0.0080 | 0.0079 | 0.0071 |
| 5 | DZ407A* | Zhengkang2 | Mihuanggu | Heibei | North summer | 0.9725 | 0.0079 | 0.0090 | 0.0106 |
| 6 | Datong29lv | . | . | Shanxi | Northwest spring | 0.0245 | 0.0071 | 0.0183 | 0.9501 |
| 7 | DZ42A* | Datong29lv | . | Heibei | North summer | 0.0231 | 0.0107 | 0.0262 | 0.9400 |
| 8 | Taixuan2 | 77-32 | Changsuihuang | Shanxi | Northwest spring | 0.0657 | 0.0412 | 0.1007 | 0.7924 |
| 9 | DZ695A* | Shi206100 | . | Heibei | North summer | 0.3352 | 0.6232 | 0.0079 | 0.0338 |
| 10 | DZ759A* | Shi206100 | . | Heibei | North summer | 0.0083 | 0.8132 | 0.1717 | 0.0068 |
| 11 | Jingu35 | Jingu14 | Qinyuanmujizui | Shanxi | Northwest spring | 0.0106 | 0.0112 | 0.9369 | 0.0413 |
| 12 | Gu621A | . | . | Heibei | North summer | 0.0098 | 0.0159 | 0.9562 | 0.0181 |
| 13 | Jingu16 | Changnong18 | Qinyuanmujizui | Shanxi | Northwest spring | 0.0314 | 0.0118 | 0.0101 | 0.9467 |
| 14 | DZ1019A* | Datong14 | . | Heibei | North summer | 0.0203 | 0.0121 | 0.0181 | 0.9495 |
| 15 | DZ207A* | Jingu29 | Qinyuanmujizui | Heibei | North summer | 0.0083 | 0.0143 | 0.9512 | 0.0262 |
| 16 | Gu367A | . | . | Heibei | North summer | 0.0069 | 0.0067 | 0.0081 | 0.9783 |
| 17 | KM402A* | Jinfen1A | . | Heibei | North summer | 0.0242 | 0.0071 | 0.0182 | 0.9505 |
| 18 | Datong14 | Xiannong3 | Xiannong3 | Shanxi | Northwest spring | 0.0690 | 0.0405 | 0.1022 | 0.7883 |
| 19 | Gu7A | . | . | Heibei | North summer | 0.0807 | 0.0074 | 0.0059 | 0.9060 |
| 20 | Gu38A | . | . | Heibei | North summer | 0.9074 | 0.0689 | 0.0062 | 0.0175 |
| 21 | KM479A* | Jin963 | . | Heibei | North summer | 0.0239 | 0.0071 | 0.0185 | 0.9505 |
| 22 | KM144A* | Gu66A | Dahuanggu | Heibei | North summer | 0.9769 | 0.0054 | 0.0071 | 0.0106 |
| 23 | KM162A* | Gu66A | Dahuanggu | Heibei | North summer | 0.9316 | 0.0114 | 0.0278 | 0.0292 |
| 24 | J512A | . | . | Heibei | North summer | 0.6361 | 0.2720 | 0.0145 | 0.0774 |
| 25 | J52A | . | . | Heibei | North summer | 0.9315 | 0.0113 | 0.0278 | 0.0294 |
| 26 | KM324A* | ZA1 | . | Heibei | North summer | 0.5493 | 0.0102 | 0.0409 | 0.3996 |
| 27 | KM306A* | ZA1 | . | Heibei | North summer | 0.8654 | 0.0121 | 0.0374 | 0.0851 |
| 28 | Gu381A | . | . | Heibei | North summer | 0.0126 | 0.0073 | 0.0144 | 0.9657 |
| 29 | KM161A* | Gu66A | Dahuanggu | Heibei | North summer | 0.9405 | 0.0349 | 0.0075 | 0.0171 |
| 30 | KM164A* | Gu66A | Dahuanggu | Heibei | North summer | 0.9477 | 0.0071 | 0.0181 | 0.0271 |
| 31 | KM168A* | Gu66A | Dahuanggu | Heibei | North summer | 0.9326 | 0.0112 | 0.0271 | 0.0291 |
| 32 | KM170A* | Gu66A | Dahuanggu | Heibei | North summer | 0.6368 | 0.2726 | 0.0144 | 0.0762 |
| 33 | KM181A* | Gu95A | . | Heibei | North summer | 0.9523 | 0.0113 | 0.0217 | 0.0147 |
| 34 | J511A | . | . | Heibei | North summer | 0.9307 | 0.0210 | 0.0323 | 0.0160 |
| 35 | KM191A* | Gu95A | . | Heibei | North summer | 0.9315 | 0.0109 | 0.0282 | 0.0294 |
| 36 | KM193A* | Gu95A | . | Heibei | North summer | 0.9526 | 0.0113 | 0.0208 | 0.0153 |
| 37 | DZ198A* | Jingu29 | Qinyuanmujizui | Heibei | North summer | 0.0432 | 0.0210 | 0.9284 | 0.0074 |
| 38 | KM200A* | Gu95A | . | Heibei | North summer | 0.9715 | 0.0096 | 0.0050 | 0.0139 |
| 39 | KM201A | Gu95A | . | Heibei | North summer | 0.8308 | 0.1439 | 0.0072 | 0.0181 |
| 40 | DZ1180A | An9217 | Riben60ri | Heibei | North summer | 0.0412 | 0.8704 | 0.0802 | 0.0082 |
| 41 | An9217 | Yugu1 | Riben60ri | Henan | North summer | 0.0481 | 0.7978 | 0.1249 | 0.0292 |
| 42 | DZ1169A* | An9217 | Riben60ri | Heibei | North summer | 0.0210 | 0.9115 | 0.0561 | 0.0114 |
| 43 | DZ854A* | Shi206100 | . | Heibei | North summer | 0.1825 | 0.7592 | 0.0069 | 0.0514 |
| 44 | Shi206100 | . | . | Heibei | North summer | 0.2180 | 0.7070 | 0.0120 | 0.0630 |
| 45 | DZ233A* | Jingu29 | Qinyuanmujizui | Heibei | North summer | 0.0282 | 0.0301 | 0.9357 | 0.0060 |
| 46 | J62A | . | . | Heibei | North summer | 0.9313 | 0.0208 | 0.0319 | 0.0160 |
| 47 | DZ244A* | Yangu12 | Moligu | Heibei | North summer | 0.0279 | 0.0300 | 0.9361 | 0.0060 |
| 48 | DZ249A* | Jingu21 | Qinyuanmujizui | Heibei | North summer | 0.0279 | 0.0302 | 0.9358 | 0.0061 |
| 49 | Gu95A | . | . | Heibei | North summer | 0.9769 | 0.0077 | 0.0080 | 0.0074 |
| 50 | DZ958A* | Chaogu14 | . | Heibei | North summer | 0.0083 | 0.9752 | 0.0105 | 0.0060 |
| 51 | DZ447A* | Chaogu14 | . | Heibei | North summer | 0.0081 | 0.9750 | 0.0109 | 0.0060 |
| 52 | Gonggu70 | Yingsuigu | Yingsuigu | Jilin | Northeast spring | 0.0596 | 0.8161 | 0.1157 | 0.0086 |
| 53 | Jigu21 | . | . | Heibei | North summer | 0.0079 | 0.0253 | 0.9528 | 0.0140 |
| 54 | Datong28 | Huangruangu | Huangruangu | Shanxi | Northwest spring | 0.0358 | 0.0240 | 0.0487 | 0.8915 |
| 55 | KM230A* | Gu95A | . | Heibei | North summer | 0.6968 | 0.2532 | 0.0136 | 0.0364 |
| 56 | Chaogu14 | . | . | Liaoning | Northeast spring | 0.0063 | 0.9690 | 0.0180 | 0.0067 |
| 57 | DZ641A* | Heng2015 | . | Heibei | North summer | 0.0484 | 0.7966 | 0.1260 | 0.0290 |
| 58 | Jinangu11 | Yugu2 | Riben60ri | Shandong | North summer | 0.0060 | 0.9791 | 0.0090 | 0.0059 |
| 59 | KM272A* | Gu138A | . | Heibei | North summer | 0.0083 | 0.0163 | 0.9128 | 0.0626 |
| 60 | Gu2A | . | . | Heibei | North summer | 0.0087 | 0.7867 | 0.1905 | 0.0141 |
| 61 | Gu3A | . | . | Heibei | North summer | 0.0111 | 0.9745 | 0.0090 | 0.0054 |
| 62 | ZA1 | . | . | Heibei | Northwest spring | 0.7797 | 0.1260 | 0.0128 | 0.0816 |
| 63 | Gu52A | . | . | Heibei | North summer | 0.0216 | 0.0489 | 0.9225 | 0.0070 |
| 64 | Gu53A | . | . | Heibei | North summer | 0.0208 | 0.0217 | 0.9465 | 0.0110 |
| 65 | Gu54A | . | . | Heibei | North summer | 0.0204 | 0.0214 | 0.9474 | 0.0108 |
| 66 | Gu71A | . | . | Heibei | North summer | 0.0127 | 0.0113 | 0.0201 | 0.9559 |
| 67 | Jingu29 | Jingu21 | Qinyuanmujizui | Shanxi | Northwest spring | 0.0305 | 0.0161 | 0.9446 | 0.0088 |
| 68 | Gu572A | . | . | Heibei | North summer | 0.9700 | 0.0125 | 0.0099 | 0.0076 |
| 69 | K359 | Gufeng2 | Riben60ri | Heibei | North summer | 0.0091 | 0.9647 | 0.0142 | 0.0120 |
| 70 | Gu12A | . |  | Heibei | North summer | 0.0065 | 0.9680 | 0.0188 | 0.0067 |
| 71 | DZ869A* | Shi206100 | . | Heibei | North summer | 0.0449 | 0.9247 | 0.0195 | 0.0109 |
| 72 | Gu56A | . | . | Heibei | North summer | 0.0183 | 0.9082 | 0.0588 | 0.0147 |
| 73 | Heng2015 | . | . | Heibei | North summer | 0.0058 | 0.8241 | 0.0151 | 0.1549 |
| 74 | KM265A* | Gu138A | . | Heibei | North summer | 0.0063 | 0.0432 | 0.9304 | 0.0201 |
| 75 | DZ972A* | Chaogu14 | . | Heibei | North summer | 0.0065 | 0.9687 | 0.0183 | 0.0065 |
| 76 | DZ1366A* | Jigu21 | . | Heibei | North summer | 0.0066 | 0.0408 | 0.9331 | 0.0195 |
| 77 | Tiedalihuang | . | . | Liaoning | Northeast spring | 0.9560 | 0.0093 | 0.0103 | 0.0244 |
| 78 | Jigu20 | . | . | Heibei | North summer | 0.0128 | 0.0071 | 0.0143 | 0.9658 |
| 79 | Yangu12 | Lvgu2 | Moligu | Shaanxi | Northwest spring | 0.0304 | 0.0157 | 0.9449 | 0.0090 |
| 80 | Gu61A | . | . | Heibei | North summer | 0.0470 | 0.0116 | 0.9306 | 0.0108 |
| 81 | Gu531A | . | . | Heibei | North summer | 0.1305 | 0.0126 | 0.0139 | 0.8430 |
| 82 | Jigu19 | Ai88 | Riben60ri | Heibei | North summer | 0.0096 | 0.9588 | 0.0249 | 0.0067 |
| 83 | DZ777A* | Shi206100 | . | Heibei | North summer | 0.4513 | 0.4991 | 0.0133 | 0.0364 |
| 84 | DZ227A* | Jingu35 | Qinyuanmujizui | Heibei | North summer | 0.0084 | 0.0173 | 0.9101 | 0.0642 |
| 85 | Gu83A | . | . | Heibei | North summer | 0.0066 | 0.0069 | 0.0081 | 0.9784 |
| 86 | Cang344 | Jigu25 | Riben60ri | Heibei | North summer | 0.2685 | 0.5922 | 0.0083 | 0.1310 |
| 87 | Gu468A | . | . | Heibei | North summer | 0.0150 | 0.3362 | 0.3563 | 0.2926 |
| 88 | Jigu22 | Ji8131 | . | Heibei | North summer | 0.0105 | 0.9358 | 0.0390 | 0.0147 |
| 89 | Gu571A | . | . | Heibei | North summer | 0.0128 | 0.0113 | 0.0198 | 0.9561 |
| 90 | Gu561A | . | . | Heibei | North summer | 0.0078 | 0.0114 | 0.0060 | 0.9748 |
| 91 | Fu12 | 1066A | Dahuanggu | Heibei | North summer | 0.8239 | 0.0534 | 0.0224 | 0.1003 |
| 92 | DZ147A* | Datong28 | Huangruangu | Heibei | North summer | 0.0168 | 0.0239 | 0.0109 | 0.9484 |
| 93 | Gu574A | . | . | Heibei | North summer | 0.9722 | 0.0079 | 0.0089 | 0.0110 |
| 94 | Gu522A | . | . | Heibei | North summer | 0.0127 | 0.0116 | 0.0193 | 0.9564 |
| 95 | Jinfen7A* | . | . | Shanxi | Northwest spring | 0.2306 | 0.0364 | 0.0350 | 0.6981 |
| 96 | Jigu27 | . | . | Heibei | North summer | 0.9580 | 0.0158 | 0.0119 | 0.0143 |
| 97 | Gu575A | . | . | Heibei | North summer | 0.0770 | 0.0208 | 0.0681 | 0.8341 |
| 98 | Yugu6 | 78γZheng4 | Mihuanggu | Henan | North summer | 0.9480 | 0.0072 | 0.0182 | 0.0266 |
| 99 | DZ461A* | Shi206100 | . | Heibei | North summer | 0.0218 | 0.9100 | 0.0570 | 0.0112 |
| 100 | Jin29A | Jingu21 | Qinyuanmujizui | Beijing | North summer | 0.0430 | 0.0198 | 0.9300 | 0.0072 |
| 101 | Gu55A | . | . | Heibei | North summer | 0.0738 | 0.0208 | 0.0683 | 0.8371 |
| 102 | Taixuan4 | 95pin10 | Qinyuanmujizui | Shanxi | Northwest spring | 0.0256 | 0.3887 | 0.1297 | 0.4559 |
| 103 | DZ1026A* | Bao182 | Riben60ri | Heibei | North summer | 0.0122 | 0.9389 | 0.0379 | 0.0110 |
| 104 | Bao182 | Zheng881407 | Riben60ri | Heibei | North summer | 0.0182 | 0.9071 | 0.0597 | 0.0150 |
| 105 | DZ955A* | Chaogu14 | . | Heibei | North summer | 0.0066 | 0.9681 | 0.0187 | 0.0066 |
| 106 | K1130 | C445 | Riben60ri | Heibei | North summer | 0.0098 | 0.9578 | 0.0255 | 0.0069 |
| 107 | Gu62A | . | . | Heibei | North summer | 0.0097 | 0.0164 | 0.9552 | 0.0187 |
| 108 | Jinfen2A* | . | . | Shanxi | Northwest spring | 0.0108 | 0.0115 | 0.9368 | 0.0409 |
| 109 | Jigu26 | . | . | Heibei | North summer | 0.0098 | 0.0164 | 0.9553 | 0.0185 |
| 110 | KM293A* | Gu220A | . | Heibei | North summer | 0.0223 | 0.0109 | 0.0262 | 0.9406 |
| 111 | DZ202A* | Jingu29 | Qinyuanmujizui | Heibei | North summer | 0.0082 | 0.0171 | 0.9101 | 0.0646 |
| 112 | Gonggu68 | Gonggu62 | . | Jilin | Northeast spring | 0.0061 | 0.0649 | 0.3734 | 0.5556 |
| 113 | Gu573A | . | . | Heibei | North summer | 0.2132 | 0.0167 | 0.0252 | 0.7448 |
| 114 | Gu11A | . | . | Heibei | North summer | 0.0091 | 0.0066 | 0.0093 | 0.9750 |
| 115 | KM112A | 208086A | . | Heibei | North summer | 0.0085 | 0.0147 | 0.9494 | 0.0274 |
| 116 | K1011 | . | . | Heibei | North summer | 0.0099 | 0.0693 | 0.8701 | 0.0507 |
| 117 | Gu66A | Dahuanggu | Dahuanggu | Heibei | North summer | 0.8295 | 0.1450 | 0.0072 | 0.0183 |
| 118 | An2491 | Ai88 | Riben60ri | Henan | North summer | 0.4112 | 0.4864 | 0.0150 | 0.0873 |
| 119 | Heng968 | Lugu5 | Riben60ri | Heibei | North summer | 0.0186 | 0.9648 | 0.0109 | 0.0057 |
| 120 | KM58A* | 208086A | . | Heibei | North summer | 0.0087 | 0.0149 | 0.9489 | 0.0275 |
| 121 | Xinggu 88 | Jingu28 | Heizhigu | Shanxi | Northwest spring | 0.0575 | 0.7949 | 0.1260 | 0.0216 |
| 122 | Shi207382 | . | . | Heibei | North summer | 0.0067 | 0.0050 | 0.0071 | 0.9812 |
| 123 | Gu629A | . |  | Heibei | North summer | 0.0238 | 0.0070 | 0.0186 | 0.9506 |
| 124 | Gufeng2 | 95307 | Riben60ri | Heibei | North summer | 0.0217 | 0.9109 | 0.0560 | 0.0114 |
| 125 | Chaogu12 | . | . | Liaoning | Northeast spring | 0.0079 | 0.0241 | 0.9541 | 0.0139 |
| 126 | Gu6A | . | . | Heibei | North summer | 0.0081 | 0.0249 | 0.9530 | 0.0140 |
| 127 | JinYA* | . |  | Shanxi | Northwest spring | 0.0237 | 0.0071 | 0.0182 | 0.9510 |
| 128 | KM221A* | Yangu12 | Moligu | Heibei | North summer | 0.0070 | 0.0233 | 0.9287 | 0.0410 |
| 129 | 368A | . | . | Heibei | North summer | 0.0209 | 0.0220 | 0.9467 | 0.0104 |
| 130 | Gu10A | . | . | Heibei | North summer | 0.0185 | 0.9647 | 0.0111 | 0.0057 |

**Table S2** Chloroplast DNA consensus primer pair sequences used in this study.

| Code | Name | Primer forward (5’-3’) | Primer reverse (5’-3’) | Tm (°C) | Product size (bp) |
| --- | --- | --- | --- | --- | --- |
| Ch-5 | OeP9-10-11-12-13 | GTCCACTCAGCCATCTCTCC | CCCGGCTAGGTACTGACCA | 55 | 253 |
| Ch-14 | OeP30 | CAGGTGTTCTGAATCAATCAATG | TTGTCTGATTCGAGGGGAAA | 55 | 157 |
| Ch-16 | OeP29 | TCCAGTTCGTAAAAAGAACCAAA | GGCAATTGAAATCTGGCAATA | 54 | 252 |
| Ch-19 | OeP22-23 | ACCTATTCGTCCCAGATCCAA | ATCGAACCCGCATCTTCTC | 55 | 353 |
| Ch-22 | OeP15 | CGGTACCAGTATTTACAATCTTTACTT | GCGATTAATCAAGTCCGACAA | 54 | 311 |
| Ch-29 | RGNMS3685 | AGCCAGAGCACGAGGTTTAT | AGCCAGAGCACGAGGTTTAT | 55 | 100 |
| Ch-30 | RGNMS2911b | TATTGAGTTAACCAGAGCACAA | TATTGAGTTAACCAGAGCACAA | 55 | 399 |
| Ch-31 | RGNMS2911a | GGTCTTAGGTTTGTGAAATGAT | GGTCTTAGGTTTGTGAAATGAT | 54 | 360 |
| Ch-32 | RGNMS1058 | AGTCTAGTCGTCCTCTTCACAT | AGTCTAGTCGTCCTCTTCACAT | 56 | 180 |
| Ch-39 | UGAtM3881 | AGATGATCTGGAGTTATCTTGG | GATTCCTCTGTCTCTGTGGTAT | 54 | 178 |

**Table S3** Mitochondrial DNA consensus primer pair sequences used in this study.

| Code | Name | Primer forward (5’-3’) | Primer reverse (5’-3’) | Tm (°C) | Product size (bp) | References |
| --- | --- | --- | --- | --- | --- | --- |
| fmt2 | *atp*9 | CCAAGTGAGATGTCCAAGAT | CTTCGGTTAGAGCAAAGCC | 50 | 279 | Duminil et al., 2002 |
| fmt5 | *ccb*256 | GGAAGTTAGCAAAGTTAGAC | TTGTTCTTAACAGCGATGGC | 56 | 528 | Duminil et al., 2002 |
| fmt6 | *ccb*452 | TATTACCAGACATAATTGGG | TATTACCAGACATAATTGGG | 50 | 1512 | Duminil et al., 2002 |
| fmt7 | *cox*1 | TTGTTACGACCACGAAGA | TCGGTGCCATTGCTGGAG | 58 | 1360 | Duminil et al., 2002 |
| fmt8 | *cox*2/1-2 | TTTTCTTCCTCATTCTKATTT | CCACTCTATTGTCCACTTCTA | 50 | 376 | Dumolin‐Lapegue et al., 1997 |
| fmt12 | *nad*2/1-2 | AATGTGGGTTGGCTTGGWTT | AATATGTAAAATTGTCCCTC | 62 | 1105 | Duminil et al., 2002 |
| fmt13 | *nad*2/3-4 | AGAAARGAATGCTGTAACCG | ATGGGGATTKTYARTATCGC | 58 | 2715 | Duminil et al., 2002 |
| fmt14 | *nad*2/4-5 | TTCATATAGAATCCATGTCC | CTATTTGTTCTTCGCCGCTT | 62 | 1800 | Duminil et al., 2002 |
| fmt15 | *nad*4/1-2 | CAGTGGGTTGGTCTGGTAATG | TCATATGGGCTACTGAGGAG | 57.5 | 2103 | Demesure et al., 1995 |
| fmt16 | *nad*4/2-3 | CTCCTCAGTAGCCCATATGA | AACCAGTCCATGACTTAACA | 55 | 3184 | Dumolin‐Lapegue et al., 1997 |
| fmt18 | *nad*4L/*orf*25 | CTGTYTTTTCGCACTTAGGC | GTCCGRGGTACTATTGCTGT | 52 | 671 | Duminil et al., 2002 |
| fmt19 | *nad*5/1-2 | TTTTTTCGGACGTTTTCTAG | TTTGGCCAAGTATCCTACAA | 57 | 2225 | Dumolin‐Lapegue et al., 1997 |
| fmt21 | *nad*6 | TGAGTGGGTCWGTCGTCCTC | TGATACTTTCTGTTTTGTCG | 58 | 605 | Duminil et al., 2002 |
| fmt24 | *nad*7/3-4 | TCTATGATGGCCCAAGAACA | ACACCAAATTCTCCTTTAGG | 47 | 3652 | Dumolin‐Lapegue et al., 1997 |
| fmt29 | *rps*3 | GGCGTATTTCGGATGCTT | TCAAGTYGGTTCAGTGAG | 50 | 2076 | Duminil et al., 2002 |
| fmt31 | *rps*12-1/ *nad*3-2/ | TTTCTTCTCTACCATGACGA | TGATCCYACTCGGTSTTCCT | 50 | 700 | Duminil et al., 2002 |
| fmt34 | *rrn*5/*rrn*18-2 | GAGGTCGGAATGGGATCGGG | CGTAASGCGTGGGAATCTGC | 58 | 1976 | Duminil et al., 2002 |

**Table S4** Classification of 130 foxtail millet accessions using 10 cpDNA consensus primers as well as the coincidence rate between the population structure and the female parent-derivative evolutionary graph.

| No. | Variety | Subpopulation membership coefficients | | | Group | Coincidence rate |
| --- | --- | --- | --- | --- | --- | --- |
|  |  | G1 | G2 | G3 |  |  |
| 85 | Gu83A | 0.9698 | 0.0140 | 0.0162 | G1 | 29.00% |
| 19 | Gu7A | 0.9671 | 0.0151 | 0.0178 |  |  |
| 94 | Gu522A | 0.9564 | 0.0136 | 0.0300 |  |  |
| 90 | Gu561A | 0.9552 | 0.0155 | 0.0293 |  |  |
| 1 | Gu51A | 0.9499 | 0.0183 | 0.0318 |  |  |
| 16 | Gu367A | 0.9482 | 0.0151 | 0.0367 |  |  |
| 42 | DZ1169A | 0.9152 | 0.0517 | 0.0331 |  |  |
| 2 | DZ234A | 0.8943 | 0.0645 | 0.0412 |  |  |
| 59 | KM272A | 0.8741 | 0.0311 | 0.0948 |  |  |
| 60 | Gu2A | 0.8597 | 0.1213 | 0.0190 |  |  |
| 28 | Gu381A | 0.8325 | 0.1348 | 0.0327 |  |  |
| 21 | KM479A | 0.8245 | 0.1120 | 0.0635 |  |  |
| 77 | Tiedalihuang | 0.8126 | 0.0845 | 0.1029 |  |  |
| 78 | Jigu20 | 0.7837 | 0.0412 | 0.1751 |  |  |
| 81 | Gu531A | 0.7660 | 0.0464 | 0.1876 |  |  |
| 65 | Gu54A | 0.7467 | 0.0192 | 0.2341 |  |  |
| 7 | DZ42A | 0.7093 | 0.0487 | 0.2420 |  |  |
| 38 | KM200A | 0.6845 | 0.2984 | 0.0171 |  |  |
| 31 | KM168A | 0.6772 | 0.1957 | 0.1271 |  |  |
| 89 | Gu571A | 0.6704 | 0.0215 | 0.3081 |  |  |
| 14 | DZ1019A | 0.6510 | 0.0911 | 0.2579 |  |  |
| 91 | Fu12 | 0.6505 | 0.0511 | 0.2984 |  |  |
| 35 | KM191A | 0.6480 | 0.1457 | 0.2063 |  |  |
| 18 | Datong14 | 0.6292 | 0.2221 | 0.1488 |  |  |
| 13 | Jingu16 | 0.6238 | 0.0511 | 0.3251 |  |  |
| 44 | Shi206100 | 0.6211 | 0.1445 | 0.2343 |  |  |
| 48 | DZ249A | 0.6078 | 0.3229 | 0.0693 |  |  |
| 25 | J52A | 0.5641 | 0.2765 | 0.1594 |  |  |
| 68 | Gu572A | 0.5623 | 0.2167 | 0.2210 |  |  |
| 127 | JinYA | 0.4791 | 0.1996 | 0.3213 |  |  |
| 87 | Gu468A | 0.4783 | 0.0558 | 0.4659 |  |  |
| 36 | KM193A | 0.4686 | 0.0578 | 0.4736 |  |  |
| 24 | J512A | 0.4510 | 0.3420 | 0.2069 |  |  |
| 43 | DZ854A | 0.3985 | 0.2036 | 0.3979 |  |  |
| 26 | KM324A | 0.4589 | 0.4959 | 0.0453 | G2 | 37.50% |
| 63 | Gu52A | 0.4464 | 0.5033 | 0.0503 |  |  |
| 54 | Datong28 | 0.4366 | 0.5381 | 0.0253 |  |  |
| 83 | DZ777A | 0.4150 | 0.4378 | 0.1473 |  |  |
| 80 | Gu61A | 0.4104 | 0.5663 | 0.0233 |  |  |
| 115 | KM112A | 0.3905 | 0.5378 | 0.0717 |  |  |
| 30 | KM164A | 0.3639 | 0.6021 | 0.0340 |  |  |
| 11 | Jingu35 | 0.3517 | 0.5938 | 0.0545 |  |  |
| 37 | DZ198A | 0.3283 | 0.5743 | 0.0974 |  |  |
| 75 | DZ972A | 0.3282 | 0.6343 | 0.0375 |  |  |
| 76 | DZ1366A | 0.2989 | 0.6724 | 0.0287 |  |  |
| 3 | KM182A | 0.2631 | 0.7180 | 0.0189 |  |  |
| 64 | Gu53A | 0.2541 | 0.7036 | 0.0423 |  |  |
| 67 | Jingu29 | 0.2435 | 0.6746 | 0.0819 |  |  |
| 108 | Jinfen2A | 0.2382 | 0.6472 | 0.1146 |  |  |
| 74 | KM265A | 0.2214 | 0.7562 | 0.0224 |  |  |
| 40 | DZ1180A | 0.2171 | 0.7416 | 0.0414 |  |  |
| 29 | KM161A | 0.1793 | 0.5928 | 0.2278 |  |  |
| 100 | Jin29A | 0.1719 | 0.6824 | 0.1457 |  |  |
| 55 | KM230A | 0.1653 | 0.7739 | 0.0608 |  |  |
| 66 | Gu71A | 0.1638 | 0.8051 | 0.0311 |  |  |
| 122 | Shi207382 | 0.1255 | 0.8357 | 0.0388 |  |  |
| 10 | DZ759A | 0.1164 | 0.6246 | 0.2590 |  |  |
| 27 | KM306A | 0.1029 | 0.7994 | 0.0977 |  |  |
| 45 | DZ233A | 0.0908 | 0.8527 | 0.0565 |  |  |
| 106 | K1130 | 0.0833 | 0.8397 | 0.0770 |  |  |
| 107 | Gu62A | 0.0806 | 0.4666 | 0.4527 |  |  |
| 12 | Gu621A | 0.0671 | 0.8907 | 0.0422 |  |  |
| 17 | KM402A | 0.0594 | 0.8364 | 0.1042 |  |  |
| 73 | Heng2015 | 0.0446 | 0.8878 | 0.0676 |  |  |
| 71 | DZ869A | 0.0378 | 0.9285 | 0.0337 |  |  |
| 113 | Gu573A | 0.0374 | 0.7522 | 0.2103 |  |  |
| 62 | ZA1 | 0.0371 | 0.7014 | 0.2615 |  |  |
| 46 | J62A | 0.0361 | 0.9130 | 0.0509 |  |  |
| 4 | DZ386A | 0.0341 | 0.8492 | 0.1167 |  |  |
| 33 | KM181A | 0.0337 | 0.9254 | 0.0409 |  |  |
| 102 | Taixuan4 | 0.0310 | 0.9305 | 0.0385 |  |  |
| 58 | Jinangu11 | 0.0310 | 0.8268 | 0.1422 |  |  |
| 104 | Bao182 | 0.0310 | 0.6896 | 0.2794 |  |  |
| 124 | Gufeng2 | 0.0286 | 0.9481 | 0.0233 |  |  |
| 117 | Gu66A | 0.0276 | 0.9315 | 0.0409 |  |  |
| 57 | DZ641A | 0.0241 | 0.5257 | 0.4502 |  |  |
| 15 | DZ207A | 0.0217 | 0.9324 | 0.0459 |  |  |
| 109 | Jigu26 | 0.0217 | 0.4968 | 0.4814 |  |  |
| 61 | Gu3A | 0.0215 | 0.9520 | 0.0265 |  |  |
| 8 | Taixuan2 | 0.0214 | 0.8870 | 0.0916 |  |  |
| 53 | Jigu21 | 0.0199 | 0.9594 | 0.0207 |  |  |
| 32 | KM170A | 0.0196 | 0.9375 | 0.0429 |  |  |
| 72 | Gu56A | 0.0190 | 0.6969 | 0.2841 |  |  |
| 49 | Gu95A | 0.0172 | 0.9345 | 0.0483 |  |  |
| 47 | DZ244A | 0.0170 | 0.9475 | 0.0355 |  |  |
| 69 | K359 | 0.0170 | 0.9463 | 0.0367 |  |  |
| 5 | DZ407A | 0.0170 | 0.9194 | 0.0636 |  |  |
| 34 | J511A | 0.0165 | 0.9497 | 0.0338 |  |  |
| 116 | K1011 | 0.0163 | 0.9411 | 0.0426 |  |  |
| 119 | Heng968 | 0.0163 | 0.6928 | 0.2909 |  |  |
| 123 | Gu629A | 0.0161 | 0.9536 | 0.0303 |  |  |
| 79 | Yangu12 | 0.0156 | 0.9305 | 0.0539 |  |  |
| 105 | DZ955A | 0.0130 | 0.7775 | 0.2095 |  |  |
| 56 | Chaogu14 | 0.0123 | 0.9550 | 0.0327 |  |  |
| 112 | Gonggu68 | 0.4003 | 0.1133 | 0.4864 | G3 | 35.72% |
| 84 | DZ227A | 0.3923 | 0.0495 | 0.5582 |  |  |
| 128 | KM221A | 0.3880 | 0.0617 | 0.5503 |  |  |
| 51 | DZ447A | 0.3340 | 0.0527 | 0.6133 |  |  |
| 92 | DZ147A | 0.3322 | 0.1095 | 0.5582 |  |  |
| 86 | Cang344 | 0.3228 | 0.0338 | 0.6434 |  |  |
| 50 | DZ958A | 0.3170 | 0.0360 | 0.6470 |  |  |
| 52 | Gonggu70 | 0.2912 | 0.0349 | 0.6739 |  |  |
| 96 | Jigu27 | 0.2899 | 0.1095 | 0.6006 |  |  |
| 126 | Gu6A | 0.2772 | 0.0397 | 0.6831 |  |  |
| 22 | KM144A | 0.2029 | 0.1407 | 0.6564 |  |  |
| 93 | Gu574A | 0.1963 | 0.0545 | 0.7492 |  |  |
| 130 | Gu10A | 0.1832 | 0.2546 | 0.5622 |  |  |
| 20 | Gu38A | 0.1764 | 0.1148 | 0.7088 |  |  |
| 99 | DZ461A | 0.1649 | 0.0445 | 0.7906 |  |  |
| 6 | Datong29lv | 0.1586 | 0.0987 | 0.7427 |  |  |
| 88 | Jigu22 | 0.1486 | 0.0724 | 0.7790 |  |  |
| 95 | Jinfen7A | 0.1373 | 0.0396 | 0.8231 |  |  |
| 114 | Gu11A | 0.1120 | 0.0465 | 0.8415 |  |  |
| 111 | DZ202A | 0.1109 | 0.0492 | 0.8399 |  |  |
| 41 | An9217 | 0.0927 | 0.1279 | 0.7795 |  |  |
| 23 | KM162A | 0.0782 | 0.0805 | 0.8413 |  |  |
| 125 | Chaogu12 | 0.0607 | 0.0716 | 0.8677 |  |  |
| 82 | Jigu19 | 0.0586 | 0.3282 | 0.6132 |  |  |
| 9 | DZ695A | 0.0553 | 0.3409 | 0.6038 |  |  |
| 118 | An2491 | 0.0505 | 0.1120 | 0.8375 |  |  |
| 121 | Xinggu88 | 0.0322 | 0.3570 | 0.6108 |  |  |
| 103 | DZ1026A | 0.0281 | 0.1514 | 0.8205 |  |  |
| 97 | Gu575A | 0.0268 | 0.0287 | 0.9445 |  |  |
| 39 | KM201A | 0.0255 | 0.1860 | 0.7885 |  |  |
| 101 | Gu55A | 0.0245 | 0.4107 | 0.5648 |  |  |
| 120 | KM58A | 0.0235 | 0.0321 | 0.9444 |  |  |
| 110 | KM293A | 0.0211 | 0.1292 | 0.8497 |  |  |
| 129 | 368A | 0.0210 | 0.0550 | 0.9240 |  |  |
| 98 | Yugu6 | 0.0203 | 0.0209 | 0.9588 |  |  |
| 70 | Gu12A | 0.0201 | 0.3811 | 0.5988 |  |  |

**Table S5** Classification of 130 foxtail millet accessions by using 17 mtDNA consensus primers as well as the coincidence rate between the population structure and the female parent-derivative evolutionary graph.

| No. | Variety | Subpopulation membership coefficients | | | Group | Coincidence rate |
| --- | --- | --- | --- | --- | --- | --- |
|  |  | G1 | G2 | G3 |  |  |
| 115 | KM112A | 0.9865 | 0.0075 | 0.0060 | G1 | 45.46% |
| 40 | DZ1180A | 0.9844 | 0.0106 | 0.0050 |  |  |
| 39 | KM201A | 0.9837 | 0.0099 | 0.0064 |  |  |
| 6 | Datong29lv | 0.9836 | 0.0083 | 0.0081 |  |  |
| 3 | KM182A | 0.9832 | 0.0124 | 0.0044 |  |  |
| 45 | DZ233A | 0.9829 | 0.0113 | 0.0058 |  |  |
| 104 | Bao182 | 0.9826 | 0.0133 | 0.0041 |  |  |
| 4 | DZ386A | 0.9821 | 0.0108 | 0.0071 |  |  |
| 43 | DZ854A | 0.9821 | 0.0105 | 0.0074 |  |  |
| 103 | DZ1026A | 0.9819 | 0.0108 | 0.0073 |  |  |
| 125 | Chaogu12 | 0.9819 | 0.0098 | 0.0083 |  |  |
| 107 | Gu62A | 0.9809 | 0.0103 | 0.0088 |  |  |
| 112 | Gonggu68 | 0.9805 | 0.0118 | 0.0077 |  |  |
| 47 | DZ244A | 0.9804 | 0.0154 | 0.0042 |  |  |
| 46 | J62A | 0.9801 | 0.0155 | 0.0044 |  |  |
| 102 | Taixuan4 | 0.9796 | 0.0142 | 0.0062 |  |  |
| 34 | J511A | 0.9789 | 0.0162 | 0.0049 |  |  |
| 42 | DZ1169A | 0.9783 | 0.0129 | 0.0088 |  |  |
| 41 | An9217 | 0.9779 | 0.0125 | 0.0096 |  |  |
| 122 | Shi207382 | 0.9757 | 0.0095 | 0.0148 |  |  |
| 38 | KM200A | 0.9746 | 0.0136 | 0.0118 |  |  |
| 118 | An2491 | 0.9746 | 0.0109 | 0.0145 |  |  |
| 21 | KM479A | 0.9743 | 0.0200 | 0.0057 |  |  |
| 36 | KM193A | 0.9741 | 0.0140 | 0.0119 |  |  |
| 119 | Heng968 | 0.9729 | 0.0124 | 0.0147 |  |  |
| 37 | DZ198A | 0.9687 | 0.0193 | 0.0120 |  |  |
| 35 | KM191A | 0.9664 | 0.0203 | 0.0133 |  |  |
| 120 | KM58A | 0.9664 | 0.0156 | 0.0180 |  |  |
| 98 | Yugu6 | 0.9655 | 0.0178 | 0.0167 |  |  |
| 116 | K1011 | 0.9652 | 0.0154 | 0.0194 |  |  |
| 114 | Gu11A | 0.9650 | 0.0086 | 0.0264 |  |  |
| 124 | Gufeng2 | 0.9638 | 0.0166 | 0.0196 |  |  |
| 126 | Gu 6A | 0.9637 | 0.0214 | 0.0149 |  |  |
| 5 | DZ407A | 0.9634 | 0.0296 | 0.0070 |  |  |
| 117 | Gu66A | 0.9593 | 0.0126 | 0.0281 |  |  |
| 53 | Jigu21 | 0.9590 | 0.0213 | 0.0197 |  |  |
| 100 | Jin29A | 0.9587 | 0.0350 | 0.0063 |  |  |
| 11 | Jingu35 | 0.9472 | 0.0443 | 0.0085 |  |  |
| 58 | Jinangu11 | 0.9459 | 0.0129 | 0.0412 |  |  |
| 44 | Shi206100 | 0.9412 | 0.0171 | 0.0417 |  |  |
| 24 | J512A | 0.9347 | 0.0171 | 0.0482 |  |  |
| 106 | K1130 | 0.9087 | 0.0445 | 0.0468 |  |  |
| 105 | DZ955A | 0.8985 | 0.0890 | 0.0125 |  |  |
| 127 | JinYA | 0.8835 | 0.0117 | 0.1048 |  |  |
| 129 | 368A | 0.8571 | 0.1239 | 0.0190 |  |  |
| 33 | KM181A | 0.7730 | 0.2230 | 0.0040 |  |  |
| 60 | Gu2A | 0.6384 | 0.0200 | 0.3416 |  |  |
| 121 | Xinggu88 | 0.6153 | 0.0491 | 0.3356 |  |  |
| 49 | Gu95A | 0.5556 | 0.0083 | 0.4361 |  |  |
| 28 | Gu381A | 0.5297 | 0.3983 | 0.0720 |  |  |
| 48 | DZ249A | 0.4455 | 0.5423 | 0.0122 | G2 | 33.33% |
| 27 | KM306A | 0.3082 | 0.5097 | 0.1820 |  |  |
| 22 | KM144A | 0.1924 | 0.8027 | 0.0049 |  |  |
| 108 | Jinfen2A | 0.1072 | 0.6792 | 0.2135 |  |  |
| 1 | Gu51A | 0.1029 | 0.8452 | 0.0519 |  |  |
| 83 | DZ777A | 0.0984 | 0.5392 | 0.3624 |  |  |
| 85 | Gu83A | 0.0938 | 0.7739 | 0.1323 |  |  |
| 26 | KM324A | 0.0930 | 0.8800 | 0.0270 |  |  |
| 95 | Jinfen7A | 0.0867 | 0.5086 | 0.4047 |  |  |
| 18 | Datong14 | 0.0822 | 0.7356 | 0.1822 |  |  |
| 20 | Gu38A | 0.0601 | 0.8968 | 0.0431 |  |  |
| 30 | KM164A | 0.0561 | 0.9056 | 0.0383 |  |  |
| 54 | Datong28 | 0.0540 | 0.6785 | 0.2675 |  |  |
| 17 | KM402A | 0.0510 | 0.9429 | 0.0061 |  |  |
| 9 | DZ695A | 0.0500 | 0.7853 | 0.1647 |  |  |
| 2 | DZ234A | 0.0454 | 0.6609 | 0.2937 |  |  |
| 12 | Gu621A | 0.0378 | 0.9499 | 0.0123 |  |  |
| 13 | Jingu16 | 0.0373 | 0.9503 | 0.0124 |  |  |
| 25 | J52A | 0.0355 | 0.8801 | 0.0844 |  |  |
| 7 | DZ42A | 0.0293 | 0.9527 | 0.0180 |  |  |
| 15 | DZ207A | 0.0292 | 0.9594 | 0.0114 |  |  |
| 56 | Chaogu14 | 0.0224 | 0.5846 | 0.3930 |  |  |
| 69 | K359 | 0.0220 | 0.5766 | 0.4014 |  |  |
| 113 | Gu573A | 0.0213 | 0.9143 | 0.0644 |  |  |
| 50 | DZ958A | 0.0213 | 0.7521 | 0.2266 |  |  |
| 51 | DZ447A | 0.0206 | 0.9596 | 0.0198 |  |  |
| 109 | Jigu26 | 0.0184 | 0.8635 | 0.1181 |  |  |
| 99 | DZ461A | 0.0174 | 0.9146 | 0.0680 |  |  |
| 63 | Gu52A | 0.0166 | 0.9253 | 0.0581 |  |  |
| 16 | Gu367A | 0.0161 | 0.9716 | 0.0123 |  |  |
| 64 | Gu53A | 0.0161 | 0.6420 | 0.3419 |  |  |
| 32 | KM170A | 0.0160 | 0.9680 | 0.0160 |  |  |
| 62 | ZA1 | 0.0141 | 0.9495 | 0.0364 |  |  |
| 101 | Gu55A | 0.0132 | 0.9808 | 0.0060 |  |  |
| 31 | KM168A | 0.0128 | 0.9789 | 0.0083 |  |  |
| 110 | KM293A | 0.0124 | 0.9565 | 0.0311 |  |  |
| 8 | Taixuan2 | 0.0115 | 0.9798 | 0.0087 |  |  |
| 65 | Gu54A | 0.0115 | 0.9058 | 0.0827 |  |  |
| 55 | KM230A | 0.0114 | 0.9341 | 0.0545 |  |  |
| 23 | KM162A | 0.0113 | 0.9808 | 0.0079 |  |  |
| 97 | Gu575A | 0.0106 | 0.9812 | 0.0082 |  |  |
| 10 | DZ759A | 0.0099 | 0.9774 | 0.0127 |  |  |
| 94 | Gu522A | 0.0083 | 0.9469 | 0.0448 |  |  |
| 66 | Gu71A | 0.0079 | 0.9153 | 0.0768 |  |  |
| 19 | Gu7A | 0.0063 | 0.9575 | 0.0362 |  |  |
| 61 | Gu3A | 0.4011 | 0.0068 | 0.5921 | G3 | 45.45% |
| 81 | Gu531A | 0.3094 | 0.0149 | 0.6757 |  |  |
| 90 | Gu561A | 0.1130 | 0.0361 | 0.8509 |  |  |
| 96 | Jigu27 | 0.0967 | 0.0068 | 0.8965 |  |  |
| 82 | Jigu19 | 0.0501 | 0.1242 | 0.8256 |  |  |
| 123 | Gu629A | 0.0446 | 0.0537 | 0.9017 |  |  |
| 14 | DZ1019A | 0.0421 | 0.0871 | 0.8708 |  |  |
| 29 | KM161A | 0.0266 | 0.2306 | 0.7428 |  |  |
| 70 | Gu12A | 0.0243 | 0.0806 | 0.8951 |  |  |
| 130 | Gu10A | 0.0222 | 0.1250 | 0.8528 |  |  |
| 111 | DZ202A | 0.0214 | 0.2715 | 0.7071 |  |  |
| 59 | KM272A | 0.0203 | 0.1564 | 0.8233 |  |  |
| 78 | Jigu20 | 0.0164 | 0.0215 | 0.9621 |  |  |
| 79 | Yangu12 | 0.0145 | 0.0146 | 0.9709 |  |  |
| 84 | DZ227A | 0.0124 | 0.2137 | 0.7739 |  |  |
| 128 | KM221A | 0.0121 | 0.0410 | 0.9469 |  |  |
| 52 | Gonggu70 | 0.0095 | 0.0217 | 0.9688 |  |  |
| 86 | Cang344 | 0.0089 | 0.0164 | 0.9747 |  |  |
| 67 | Jingu29 | 0.0087 | 0.0197 | 0.9716 |  |  |
| 87 | Gu468A | 0.0085 | 0.0169 | 0.9746 |  |  |
| 57 | DZ641A | 0.0081 | 0.0185 | 0.9734 |  |  |
| 75 | DZ972A | 0.0081 | 0.0122 | 0.9797 |  |  |
| 88 | Jigu22 | 0.0079 | 0.0344 | 0.9577 |  |  |
| 71 | DZ869A | 0.0067 | 0.2371 | 0.7562 |  |  |
| 91 | Fu12 | 0.0064 | 0.0076 | 0.9860 |  |  |
| 76 | DZ1366A | 0.0060 | 0.0162 | 0.9778 |  |  |
| 72 | Gu56A | 0.0060 | 0.0095 | 0.9845 |  |  |
| 74 | KM265A | 0.0058 | 0.0121 | 0.9821 |  |  |
| 68 | Gu572A | 0.0058 | 0.0092 | 0.9850 |  |  |
| 93 | Gu574A | 0.0050 | 0.0063 | 0.9887 |  |  |
| 73 | Heng2015 | 0.0049 | 0.0061 | 0.9890 |  |  |
| 80 | Gu61A | 0.0049 | 0.0060 | 0.9891 |  |  |
| 77 | Tiedalihuang | 0.0044 | 0.0069 | 0.9887 |  |  |
| 89 | Gu571A | 0.0043 | 0.0076 | 0.9881 |  |  |
| 92 | DZ147A | 0.0040 | 0.0059 | 0.9901 |  |  |

**Fig. S1** Population structure analysis for 130 accessions of foxtail millet by using 10 cpDNA consensus primers. (A) Delta *K* values for different numbers of populations assumed (*K*) in the structure analysis. (B) Classification of 130 accessions into three subpopulations according to preset *K* value using the STRUCTURE program. The distribution of the accessions among different subpopulations is represented by color (G1: red, G2: blue; G3: green).

**Fig. S2** Population structure analysis for 130 accessions of foxtail millet by using 17 mtDNA consensus primers. (A) Delta *K* values for different numbers of populations assumed (*K*) in the structure analysis. (B) Classification of 130 accessions into three subpopulations according to preset *K* value using the STRUCTURE program. The distribution of the accessions among different subpopulations is represented by color (G1: red, G2: blue; G3: green).
